# A population-based study of human immunodeficiency virus in south India reveals major differences from sentinel surveillance-based estimates

**DOI:** 10.1186/1741-7015-4-31

**Published:** 2006-12-13

**Authors:** Lalit Dandona, Vemu Lakshmi, Talasila Sudha, G Anil Kumar, Rakhi Dandona

**Affiliations:** 1 Health Studies Area, Centre for Human Development, Administrative Staff College of India, Hyderabad, India; 2 Department of Microbiology, Nizam's Institute of Medical Sciences, Hyderabad, India

## Abstract

**Background:**

The human immunodeficiency virus (HIV) burden among adults in India is estimated officially by direct extrapolation of annual sentinel surveillance data from public-sector antenatal and sexually transmitted infection (STI) clinics and some high-risk groups. The validity of these extrapolations has not been systematically examined with a large sample population-based study.

**Methods:**

We sampled 13838 people, 15–49 years old, from 66 rural and urban clusters using a stratified random method to represent adults in Guntur district in the south Indian state of Andhra Pradesh. We interviewed the sampled participants and obtained dried blood spots from them, and tested blood for HIV antibody, antigen and nucleic acid. We calculated the number of people with HIV in Guntur district based on these data, compared it with the estimate using the sentinel surveillance data and method, and analysed health services use data to understand the differences.

**Results:**

In total, 12617 people (91.2% of the sampled group) gave a blood sample. Adjusted HIV prevalence was 1.72% (95% confidence interval 1.35–2.09%); men 1.74% (1.27–2.21%), women 1.70% (1.36–2.04%); rural 1.64% (1.10–2.18%), urban 1.89% (1.39–2.39%). HIV prevalence was 2.58% and 1.20% in people in the lower and upper halves of a standard of living index (SLI). Of women who had become pregnant during the past 2 years, 21.1% had used antenatal care in large public-sector hospitals participating in sentinel surveillance. There was an over-representation of the lowest SLI quartile (44.7%) in this group, and 3.61% HIV prevalence versus 1.08% in the remaining pregnant women. HIV prevalence was higher in that group even when women were matched for the same SLI half (lower half 4.39%, upper 2.63%) than in the latter (lower 1.06%, upper 1.05%), due to referral of HIV-positive/suspected women by private practitioners to public hospitals. The sentinel surveillance method (HIV prevalence: antenatal clinic 3%, STI clinic 22.8%, female sex workers 12.8%) led to an estimate of 112635 (4.38%) people with HIV, 15–49 years old, in Guntur district, which was 2.5 times the 45942 (1.79%) estimate based on our population-based study.

**Conclusion:**

The official method in India leads to a gross overestimation of the HIV burden in this district due to addition of substantial extra HIV estimates from STI clinics, the common practice of referral of HIV-positive/suspected people to public hospitals, and a preferential use of public hospitals by people in lower socioeconomic strata. India may be overestimating its HIV burden with the currently used official estimation method.

## Background

It has been suggested recently that India now has the highest number of people living with human immunodeficiency virus (HIV) in the world [1]. India's national organisation for control of acquired immunodeficiency disease syndrome (AIDS), the National AIDS Control Organization (NACO), uses public-sector sentinel surveillance data to estimate the HIV burden annually [2,3]. However, the validity of arriving at population estimates from direct extrapolation of the sentinel surveillance HIV data, as is currently done in India, has not yet been systematically examined [3,4]. A reliable estimate of HIV burden is critical, as it is the first step towards informed planning of HIV control including treatment requirements. The importance of HIV estimates is highlighted by the marked attention received by the Joint United Nations programme on AIDS (UNAIDS) estimates for countries around the world released every 2 years, and the extensive work carried out on HIV estimation by the UNAIDS reference group on estimates, modelling and projections [1,5]. However, research to improve HIV estimates has so far been carried out predominantly in sub-Saharan Africa. Primary research to inform accurate HIV estimation in India has been sorely missing [4]. Consequently, UNAIDS estimates the plausibility range of the HIV burden for India to be very wide: 3.4–9.4 million for 2005 [1]. Clearly, a three-fold plausibility range is not optimum for informed planning of HIV control in India.

A large annual sentinel surveillance is carried out in India in the third quarter of each year, which includes data predominantly from public-sector antenatal clinics and sexually transmitted infection (STI) clinics, and also from some high-risk groups [2,3,6]. In 2005, this included 703 sites and HIV testing on 225600 people [2]. In principle, the total number of adults with HIV in each Indian state is estimated by adding the following: (i) adults in the general population having HIV (estimated by applying the median HIV prevalence from antenatal clinics, mostly at medical colleges and district headquarter hospitals, more or less directly to the adult population); (ii) adults with STIs having HIV (estimated by applying the median HIV prevalence from STI clinics, mostly at medical colleges and district headquarter hospitals, to 5–6% of the adult population assumed to get STIs every year); and (iii) high-risk groups having HIV (estimated by applying the HIV prevalence from some sites for female sex workers, men who have sex with men, and intravenous drug users to their estimated total number not covered by the preceding STI component) [6]. Application of this method to the south Indian state of Andhra Pradesh, which is estimated to have the highest HIV burden of all states in India, resulted in an estimate of 1.45 million people, 15–49 years of age, with HIV in 2005 according to NACO (Table 1).

Two previously published studies have attempted comparison of population-based HIV prevalence with public-sector antenatal clinic sentinel prevalence in India; the first in a sample of 1981 people from three districts in the state of Tamil Nadu [7], and the second in a sample of 2870 people from a district of Tamil Nadu [8]. However, the sample sizes of these studies were too small for reliable comparison with the sentinel surveillance antenatal HIV prevalence of 1% in Tamil Nadu, and comparison of the total HIV burden estimate from population-based data with that using the complete official sentinel surveillance method was not attempted. No large population-based study of HIV distribution that has systematically assessed the validity of estimating HIV burden with the currently used sentinel surveillance method has been reported from India [3,4]. Apart from this critical deficiency in the evidence base for HIV in India, a detailed assessment of the dynamics of risk factors for HIV in the general population, needed to plan informed HIV prevention strategies, has also not been reported from any large population-based study in India. To address these deficiencies, we conducted a population-based study of HIV distribution and its risk factors in Guntur district in the state of Andhra Pradesh. According to the sentinel surveillance data, Andhra Pradesh, with a population of 80 million in 2005, is estimated to have the highest burden of HIV among Indian states, and Guntur district is estimated to have one of the highest prevalence in this state [2,9].

In this study, we compared the population-based HIV estimates from this study with those obtained using the official sentinel surveillance method to identify issues that may need to be addressed to make the HIV estimation process in India more accurate.

## Methods

### Population-based sample and procedures

**Figure 1:**
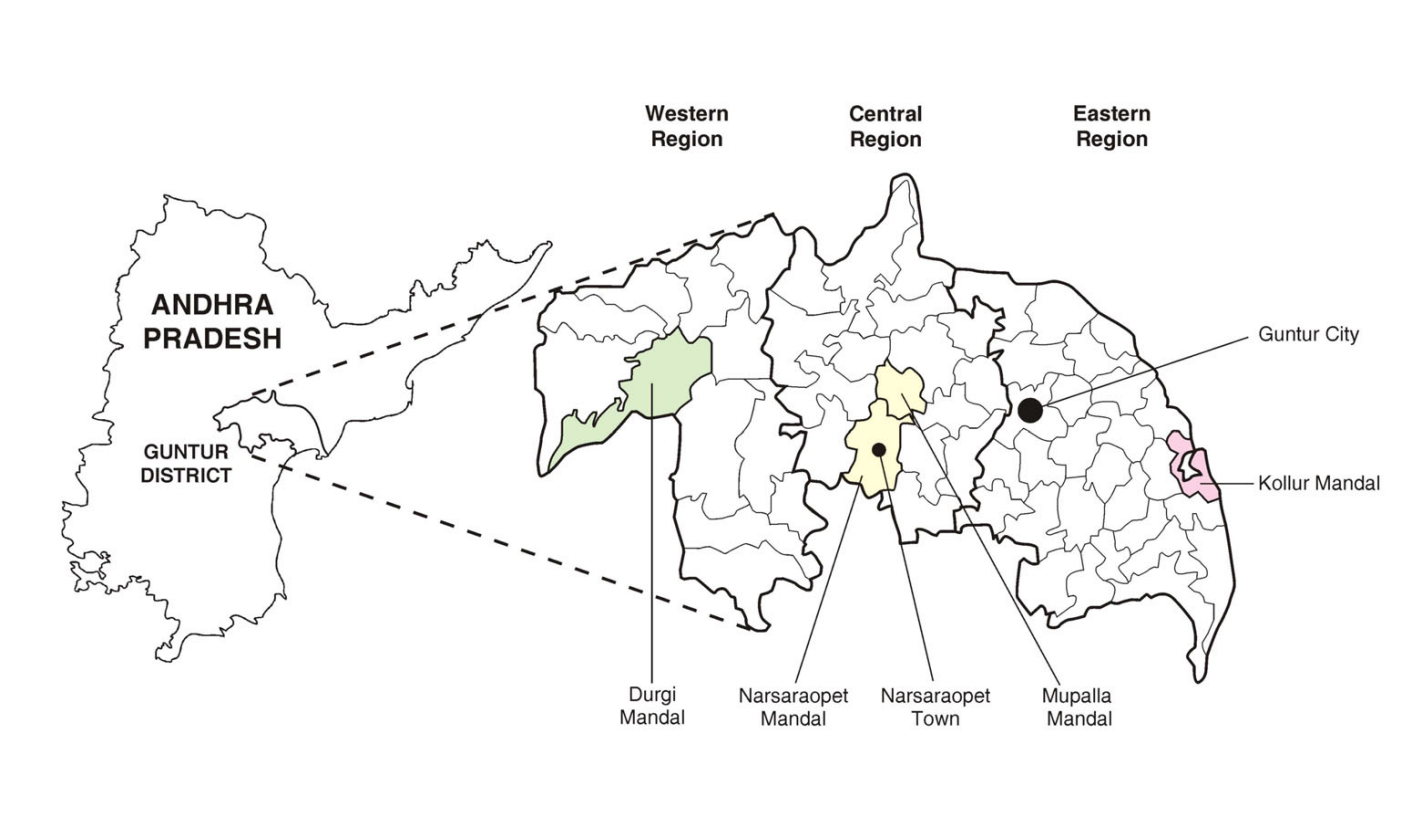
Locations sampled in Guntur district for the population-based study.

Guntur district in Andhra Pradesh had a population of 4.46 million in the 2001 census, with 29% urban [10]. The district is divided into 57 mandals (administrative units), of which 45 have a totally rural population. Urban areas include two class I urban cities with populations > 100000, and 10 smaller towns. For the purpose of sampling, we divided the district into three geographic regions of approximately equal area that had broadly similar distributions of development indicators (literacy, assets, and electricity and water access) by census data [10,11] (Figure 1).

Of the three regions, the eastern region, with a river delta and fertile land, has the highest levels of development indicators and population density, and the western region the lowest. For the urban sample, we selected Guntur city (population 514500) to represent the class I urban cities, and Narsaraopet town (population 95300) to represent the smaller towns, as these had the largest population contribution to their respective urban categories, and also because these two urban areas have antenatal clinics that provide data for sentinel surveillance, which would enable comparisons between estimates from the population and those from the antenatal clinics. For the rural sample, we initially selected one rural mandal in each of the three geographic regions in which the three development indicators were closest in combination to the rural median for the respective region, taking into account that the selected rural mandal should not be immediately next to either of the two class I cities in the district to avoid direct urban influence of large cities. To this, we added the rural portion of Narsaraopet mandal in the central region, with the intention of comparing the combined rural/urban data from Narsaraopet with the antenatal clinic data from Narsaraopet town. The populations of the four selected rural mandals were: Durgi 44600, Mupalla 41 500, Narsaraopet 84300, and Kollur 55900.

Based on census data, we divided Guntur city into 365 clusters and Narsaraopet town into 66 clusters of mostly 1300–1600 population and categorized them into lower, middle and upper socioeconomic strata. We then randomly selected 24 clusters in Guntur city and 8 in Narsaraopet town such that the proportion of selected clusters in each socioeconomic strata were similar to their proportion among the total clusters in each of these two urban areas. We also estimated the homeless population in Guntur city and Narsaraopet town and selected the largest homeless cluster in each to include 1% of the urban sample to represent the homeless. In the selected rural mandals, we divided large villages into segments of 1300–1600 population each, and combined villages with smaller population with others to make a cluster size of 1300–1600 population each, resulting in 30–60 clusters in the four mandals. We then randomly selected eight clusters in each of the four mandals.

Within each cluster (other than the two homeless clusters) we enumerated the households and residents. A household was defined as people eating from the same kitchen. A resident was defined as a person living in that city, town or village for the past 6 months or more; this minimum residence period was used to allow some time to participate in the HIV dynamics of the sampled areas. All residents 15–49 years of age in the selected cluster were considered eligible. Systematic sampling, with the first number drawn randomly, was carried out to sample households with the aim of sampling 200–230 eligible people in each cluster. This usually required a sampling interval between 3 and 5 for households, depending on the total number of households and eligible people in a cluster. All 15–49-year-old people in the selected households were considered sampled for the study. The first 44 homeless 15–49-year-olds in the selected homeless cluster in Guntur city and the first 16 in Narsaraopet town were sampled.

With this sampling approach, about 13800 people 15–49 years of age would be sampled for the study in 66 clusters. Assuming a participation rate of 90% of the eligible people, based on our pilot studies, we estimated a sample participation of about 12400 people, approximately equal men/women and rural/urban. At the time of planning this study in mid-2004, the most recent sentinel surveillance of 2003 had shown a HIV prevalence of 3.75% (15/400) at the Guntur antenatal clinic, which is one of the 23 clinics at medical colleges or district headquarter hospitals in Andhra Pradesh from which data are used by NACO for making population estimates, and the HIV prevalence among all 8870 women who attended this antenatal clinic and received prevention of mother to child transmission (PMTCT) HIV services (86.1% of all new antenatal registrations) during the most recent annual cycle 2003–04 was 3.07% (95% confidence interval [CI] 2.71–3.43%). As this PMTCT prevalence was based on a much large sample size than the annual antenatal sentinel surveillance, we used a 3% antenatal HIV prevalence to calculate for reliable comparison the sample size needed in the population-based study. Assuming a cluster design effect of 2.5, the anticipated sample size of 6200 women in our study would have 87% power to detect a 25% difference from the antenatal HIV prevalence at the 95% confidence level, and the anticipated total sample size of 12400 in our study would have 93% power to detect a 20% difference from the antenatal HIV prevalence at the 95% confidence level [12,13].

Data were collected in the rural clusters during September 2004–February 2005, and in the urban clusters during March-September 2005. Trained field investigators obtained informed consent from eligible people for participation in the study, followed by confidential interview that included demographic data, detailed history of risk factors and history of relevant health-service use. As part of the demographic data, we administered a standard of living index (SLI) based on living conditions and ownership of assets, which was adapted from an index used previously by the National Family Health Survey in India [14]. A blood sample from each respondent was obtained on filter paper (Whatman No. 3; Whatman International Ltd, Maidstone, Kent, UK) by the finger-prick method, preferably six drops, which were allowed to dry. These dried blood spots were stored in sealed polythene bags with desiccant in the field office at room temperature for a maximum of 1 week, and were transported weekly to the laboratory in Hyderabad. Because the HIV test results would be unlinked to respondent identity, those interested in knowing their HIV status were referred to the nearest voluntary counselling and testing centre.

At least five attempts were made to reach all eligible people, including visits at a later time for those who were travelling during the initial round of data collection in a particular cluster. Basic demographic data, including occupation, and the reason for non-participation were documented for those who did not participate in the study.

Ethics approval for this study was obtained from the institutional ethics committees of the Administrative Staff College of India and Nizam's Institute of Medical Sciences, Hyderabad, India. This study complied with the principles expressed in the Declaration of Helsinki.

### Laboratory methods

On arrival at the laboratory, the dried blood samples were stored under refrigeration at 2–8°C until testing for HIV was performed. The testing strategy was aimed at detecting HIV antibody (which appears about 3 weeks after infection), p24 antigen (which appears during the second or third week after infection), and viral nucleic acid (which appears during the first week after infection) [15-17]. A 6-mm punch of the dried blood spot was eluted overnight with 0.15 M phosphate-buffered saline to obtain the sample for testing. All 12617 samples were initially tested for HIV antibody or antigen by a fourth-generation ELISA (Murex HIV Combi Assay; Murex Biotech, Dartford, UK). The positive samples were tested by a third-generation ELISA (Murex HIV; Murex Biotech) to confirm the presence of HIV antibody, and those that tested positive were re-tested with a third-generation rapid HIV test (HIV Tridot; J. Mitra, New Delhi, India) to distinguish between HIV-1 and HIV-2 antibodies. The samples negative with the third-generation ELISA were tested with another fourth-generation ELISA (Vidas HIV Duo Ultra; bioMérieux, Marcy-l'Etoile, France) and an antigen-specific kit (Vidas HIV p24; bioMérieux) to confirm the presence of p24 antigen. Of the samples negative for both antibody and antigen, a subset of 585 samples belonging to people who were considered at relatively high risk of HIV (men or women with a HIV-positive spouse who had last had sex with the spouse within the past 15 days, men who had had sex with a female sex worker in the past 6 months and had last had sex with a woman within the past 15 days, identified female sex workers, men who had had sex with a man in the past 15 days, and men or women with current multiple sex partners and who had last had sex within the past 15 days) underwent qualitative PCR (Amplicor 1.5; Roche Molecular Diagnostics, Branchburg, USA) testing in pools of 10 samples for HIV viral nucleic acid to detect very recent infections. Pooling of samples as high as 96–100 for detecting HIV nucleic acid has been reported not to lead to significant loss of sensitivity [18,19].

A systematic random sample of 10% of all samples negative for HIV antibody, antigen or nucleic acid (1238) underwent quality-assurance testing. This was carried out by repeating the fourth-generation ELISA (Murex) for individual samples and PCR (Amplicor) for 10-sample pools. All retested samples remained negative with both these tests.

To determine comparability of detecting HIV from venous blood and dried blood samples, we collected both types of samples from 225 people known to have HIV and tested them in our laboratory with the fourth-generation ELISA (Murex). All samples of venous blood and dried blood samples tested positive for HIV. The literature has also reported very high sensitivity for detection of HIV from dried blood spots by ELISA and PCR compared with serum from venous blood [20,21].

The time lag between collection of blood samples and the initial ELISA testing in the laboratory was a maximum of 2 months, with the majority tested within 3 weeks. All other testing on samples stored at 2–8°C was completed within 20 months of sample collection. Previous reports have indicated no significant loss of sensitivity for detecting HIV from dried blood spots stored under refrigeration for up to 20 weeks and 36 months for ELISA and PCR, respectively [21,22].

### Sentinel surveillance and related data

We obtained HIV data from the 2005 sentinel surveillance in Andhra Pradesh carried out at antenatal clinics, STI clinics, and sites for female sex workers and men who have sex with men, for comparison with our population-based data. We obtained the method used by NACO for estimating HIV burden based on sentinel surveillance data. HIV testing in the sentinel surveillance, and in PMTCT services at antenatal clinics, is carried out with a combination of third-generation ELISA and rapid tests.

In order to determine the profile of the users of the two sentinel surveillance antenatal clinics in Guntur district, we obtained demographic data on and administered the SLI to 487 and 402 consecutive antenatal clinic attendees at Guntur and Narsaraopet, respectively, during a fixed time interval aimed at obtaining a sample of at least 400 at each clinic. We also assessed the referral pattern to these clinics in this sample.

### Statistical analysis

Statistical analyses were carried out using SPSS software (SPSS Inc, Chicago, IL, USA). The HIV estimate in each cluster, except the two homeless clusters, was age-standardized separately for both sexes with the age distribution in the rural and urban populations of Guntur district [23]. For each rural mandal, the HIV estimate in each of the eight clusters was given equal weight. For Guntur city and Narsaraopet town, HIV estimates in the homeless were given weights equal to their estimated proportion in the population (Guntur: men 0.7%, women 0.3%; Narsaraopet: men 0.5%, women 0.2%), and the other clusters were given equal weight for the non-homeless population. For composite rural HIV prevalence, the weights given to the estimates from rural mandals were equal to the proportion of total district rural population in their respective regions: 0.19 for Durgi mandal in Western region, 0.31 for Mupalla and Narsaraopet mandals combined in the central region, and 0.50 for Kollur mandal in the eastern region. For composite urban HIV prevalence, the estimate for Guntur city was given weight equal to the proportion of total district urban population in class I cities (0.52), and the estimate for Narsaraopet town was given weight equal to the proportion of total urban population in towns with < 100000 population in the central and eastern regions (0.40). The remaining 8% urban population was present in two small towns in the relatively less developed western region, which we felt could not be represented by data from the more developed Narsaraopet town. We estimated the HIV prevalence for these two towns indirectly as 1.2 times the rural prevalence in the western region, which was then given a weight of 0.08 for the composite urban HIV prevalence in Guntur district. At each level, the men-women composite rates were adjusted for the sex distribution in the population. We included the six transgender people in our sample in the men category for analysis, as separate analysis for this small number was not feasible. The 95% confidence intervals of HIV prevalence estimates were calculated taking into account the design effect of the cluster sampling strategy [24].

We compared HIV prevalence between people in the various strata of the SLI used in this study and between age groups in both sexes. As the currently used method of estimating HIV burden in India is based on sentinel surveillance data predominantly from public (government) hospitals, we compared HIV prevalence between users of public hospitals versus others in our population-based sample.

Our population-based study did not include residential hostels for young adults in urban areas or the prison population. Because residents of these facilities may be at higher risk of HIV, we estimated the total number of male and female residents in these facilities in Guntur district, and assumed that the HIV prevalence in prisoners (mostly men) was five times the prevalence in urban men and the HIV prevalence in men and women in residential hostels was twice the prevalence in the respective sexes in the general urban population. Compared with the adult men:women ratio in the Guntur district census data [23], we ended up having an under-representation of men in the eligible sample; we assumed that these missing men had twice the prevalence of HIV found in men in our sample.

We estimated the total number of adults with HIV in Guntur district by applying our population-based HIV prevalence to the 15–49-year-old men and women in the year 2005 (estimated assuming an annual growth rate of 1.37% since the 2001 census [25]), and adding to this the estimated number of people with HIV in residential hostels and prisons and among undersampled men, and also female sex workers with HIV estimated to be inadequately represented in our population-based sample (based on their estimated number in Guntur district and HIV prevalence among them).

We then estimated the total number of 15–49-year-olds with HIV in Guntur district using the sentinel surveillance method used by NACO, and compared this with the estimate based on our population-based study to identify differences and the reasons for these. We performed sensitivity analysis to assess the plausible range of the ratio between the estimates from the two methods. Based on the population-based estimates, we calculated a correction factor for sentinel surveillance data to reflect more closely the population HIV burden.

## Results

### Sample structure

**Figure 2:**
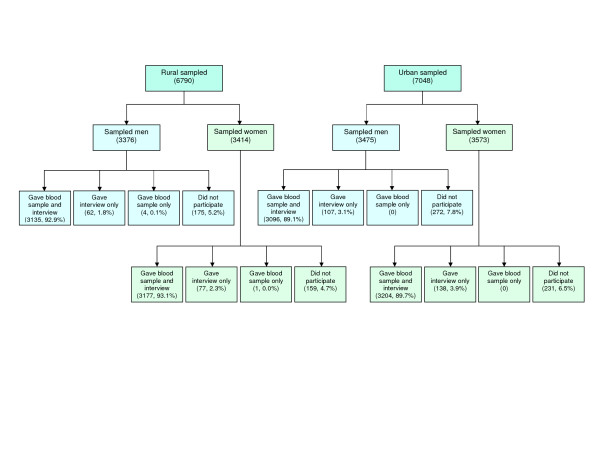
Participation profile in the population-based study.

Of the 13838 15–49-year-olds sampled, 12617 (91.2%) gave a blood sample, which included 6317 rural residents (50.1%) and 6382 women (50.6%) (Figure 2). We had undersampling of urban men by 3.7% and rural men by 3.5%, compared with their ratio to women in the census data for 15–49-year-olds in Guntur district [23]. The participation rate was slightly lower in the urban sample (89.4%) than in the rural sample (93%), but was similar for both sexes in these two samples. The participation rates were not very different across age groups both among men (ranging between 89% in the 40–44-year age group and 92.4% in the 20–24-year age group) and women (ranging between 89.4% in the 15–19-year age group and 93.4% in the 25–29-year age group). People with transport-related occupations, unskilled labourers and other occupations involving regular mobility, who may be at higher risk of HIV, had participation rates of 91.3%, 92.1% and 92.8%, respectively, which were similar to the average participation rate of 91.2%. The participation rates for different marital statuses, which may have a bearing on HIV risk, were not very different within each sex: 91.1%, 90.9% and 93.2% for men who were single, married and separated/divorced/widowed, respectively and 89.2%, 91.5% and 93.2% for the same groups of women.

Some of the other groups at relatively higher risk of HIV that were represented in the sample included men who had ever visited female sex workers (1168; 18.7% of men), identified female sex workers (9; 0.14% of women), and men who had had sex with men (132; 2.1% of men) including men who had sold sex to men (16; 0.26% of men).

As we did not stratify our rural sample for socioeconomic strata, comparison of our sample with census data for schedule caste and schedule tribes, a surrogate measure for lower socioeconomic strata, revealed that our sample had a higher proportion of this group (32.3%) than in the 2001 census of Guntur district (26.1%) [10].

### HIV distribution in the population

**Figure 3:**
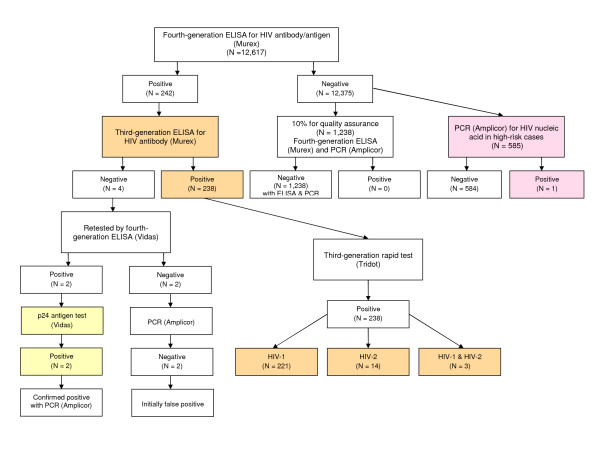
Results of laboratory testing for HIV.

In total, 241 people in the sample were found to be HIV-positive, of which two were only antigen-positive and one was positive only for nucleic acid on PCR testing (Figure 3). Of the 238 people with HIV antibody detected, the rapid test for HIV-1/HIV-2 antibodies revealed that 221 (92.9%) were positive for HIV-1, 14 (5.9%) for HIV-2, and 3 (1.3%) for both HIV-1 and HIV-2.

The overall HIV prevalence for 15–49-year-old adults in Guntur district, adjusted for age, sex and rural/urban distribution of the population in this district, was 1.72% (95% CI 1.35–2.09%). HIV prevalence was not significantly different between rural and urban areas, or between men and women, though the prevalence was slightly higher for urban areas as a whole, due to the trend towards higher prevalence in urban men (Table 2). The western region of Guntur district had a trend towards lower HIV prevalence than in the other two regions, but this was not statistically significant with the sample in this study.

Crude HIV prevalence was 4.88% (57/1168) in men who had ever visited female sex workers, 11.1% (1/9) in the few identified female sex workers, 7.58% (10/132) in men who had had sex with men, 25% (4/16) in men who had sold sex to men, and 20% (12/60) in homeless people.

The prevalence of HIV in people up to the 50th percentile of the SLI was 2.58%, over twice the 1.20% prevalence in people in the upper half of this index (Table 3). The trend of higher HIV prevalence in people with a lower SLI was seen in both rural and urban areas, as well as in both sexes. The ratio of HIV prevalence in the lowest quartile of the index to that in the highest quartile was higher in the urban (3.47) than in the rural sample (2.37). The HIV gradient for the SLI quartiles was least in rural men (Table 3).

The distribution of HIV prevalence by age showed a trend towards higher prevalence among women < 30 years of age than in the same age group of men (Table 4). The HIV prevalence among women was highest in those in their 20s compared with the highest prevalence among men in their 30s. The HIV prevalence difference between men and women was most striking in the 30–39-year age group for the urban sample, with the prevalence among men 2.4 times that in women. There was a suggestion of an earlier start of HIV among rural than among urban women, with 1.34% versus 0.35% prevalence in the 15–19-year age group, but this difference was not statistically significant with the relatively small sample size in each age group.

### HIV distribution versus type of health services used

In this sample, 777 women reported being pregnant at present or during the past 2 years. Of these, data were available for 722 regarding the type of antenatal services they used, which included private-sector services by 512 (70.9%), public-sector services in government institutions by 166 (23%) [including the category of hospitals covered by sentinel surveillance; used by 152 (21.1%)], checkup at home by 8 (1.1%) and no antenatal checkup by 36 (5%). The overall HIV prevalence in women pregnant over the past 2 years was 1.67% (13 of 777), similar to the population prevalence of 1.70% in 15–49-year-old women. However, HIV prevalence in women who used public-sector antenatal care in the category of hospitals covered by sentinel surveillance was 3.95% (6 of 152), 3.8 times the prevalence in women who used other antenatal care options (1.05%, 6 of 570); this differential was similar for both rural and urban samples. The use of public-sector antenatal care services at the category of hospitals covered by sentinel surveillance was disproportionately higher by women with a lower SLI, with 44.7% in the lowest quartile of this index and only 6.6% in the highest quartile, compared with an approximately equal distribution in the index quartiles for those who used other options for antenatal care (Table 5). Interestingly, for the same half of the SLI (lower or upper), women who used public-sector antenatal care at the category of hospitals covered by sentinel surveillance had a HIV prevalence several times higher than those who used other options for antenatal care (Table 6).

As a surrogate measure for the type of health services used for STIs, the health services used most frequently for any type of illness by men and women, respectively, were: public-sector 7.9% and 11.9%, private provider 80.5% and 78.3%, medicines from pharmacy 9.1% and 8.5%, and others 2.4% and 1.1%. The HIV prevalence in men and women who used public-sector health services was 4.49% and 3.02%, compared with 1.88% and 1.57%, respectively, in those who preferred other health-service options. Of the men and women using public-sector services, 37.3% and 41.9%, respectively, belonged to the lowest quartile of the SLI, whereas those who preferred other options were almost equally distributed in the four quartiles of the SLI. As with antenatal care, for the same half of the SLI, both men and women who used public-sector general health services had substantially higher HIV prevalence than those who preferred other options (Table 6).

### Patient profile at sentinel surveillance clinics

The distribution of SLI in a sample of the attendees in the two public-sector antenatal clinics in Guntur district that participate in the sentinel surveillance showed a disproportionately higher representation of those with lower index scores, with 47.8% and 56.7% in the lowest quartile of the index (based on our population-based distribution) at the Guntur city and Narsaraopet town clinics, respectively (Table 7). This trend in SLI distribution was similar to that of women in our population-based sample who had used antenatal care services in the public-sector hospitals during the past 2 years (Table 5).

At the Guntur and Narsaraopet antenatal clinics, 36.6%% and 34.8% of the total attendees had previously visited a private health facility for that pregnancy, and 15.7% and 20.3% of the rural attendees, and 6.3% and 8.8% of the urban attendees, respectively, had been referred by the private facility to a public-sector hospital (Table 8). The reasons given for this referral at the Guntur clinic included a HIV-positive blood test result in three women (0.62% of the total 487 sample) and the need for an unspecified blood test in nine women (1.85% of the total sample). At the Narsaraopet clinic, the reasons included the need for a HIV test in three women (0.75% of the total 402 sample) and the need for an unspecified blood test in 31 women (7.71% of the total sample).

### HIV in sentinel surveillance

Analysis of the age distribution of women who were included in the 2005 sentinel surveillance for antenatal clinics in Andhra Pradesh showed that 76% were in the 15–24-year-old age group, compared with 35.3% among the 15–49-year-old women in Andhra Pradesh [23]. The mean crude prevalence of HIV in the sentinel surveillance clinics for the 23 medical college or district headquarter clinics and 21 community health centre clinics in Andhra Pradesh were 2% and 1.32%, respectively. Adjustment for the age distribution of 15–49-year-old women in Andhra Pradesh did not change the former prevalence but slightly increased the latter to 1.54%.

During the 2005 sentinel surveillance, the HIV prevalence at the Guntur city antenatal clinic, which is one of the 23 medical college or district headquarter surveillance clinics in Andhra Pradesh from which data are used by NACO for estimation of HIV burden, was 3% (12/400). During the period of field data collection for our population-based study (September 2004–September 2005), 10838 women registered new at the Guntur antenatal clinic, of whom 10504 (96.9%) received HIV testing as part of the PMTCT services and 310 (2.95%, 95% CI 2.63–3.27%) were HIV-positive. This prevalence was significantly higher than the prevalence in our population-based study for either men or women (Table 2).

The HIV prevalence during the sentinel surveillance of 2005 at the Narsaraopet town antenatal clinic, which is one of the 21 community health centre surveillance clinics in Andhra Pradesh (data from these clinics are not used by NACO for HIV burden estimation), was 2.5% (10/400). The combined HIV prevalence in the Narsaraopet rural and urban samples in our population-based study, adjusted for age, sex and rural/urban distribution of population of Narsaraopet, was 1.92% (95% CI 1.23–2.61%; design effect 1.98), which was lower than the antenatal clinic prevalence, but with the given sample size this difference was not statistically significant. Of the 402 Narsaraopet antenatal clinic attendees from which we collected demographic data, 69.8% were from places other than rural or urban Narsaraopet, mostly nearby mandals of Guntur district. PMTCT data from Narsaraopet antenatal clinic were not available for the duration of our study, as this service was started at this clinic in late 2005.

At the 11 STI clinics in Andhra Pradesh providing data for sentinel surveillance, most of which are located at medical colleges or district headquarter hospitals, the median HIV prevalence in the 2005 surveillance round, using a sample size of 250 in each clinic, was 22.8%. There was no surveillance clinic in Guntur district, and the prevalence at the nearest clinic in Vijaywada city, very close to this district, was 26.4%. We used the more conservative prevalence of 22.8% for our calculations for Guntur district. The HIV prevalence in female sex workers during the 2005 surveillance round at the site in Guntur district was 13.2% (33/250), and the median for the 7 sites in Andhra Pradesh was 12.8%. The number of female sex workers in Guntur district in 2005 was estimated as 11000, based on a previous estimate of 10400 in 2003–04 [9]. The HIV prevalence in 2005 at the single sentinel surveillance site in Andhra Pradesh for men who have sex with men was 6.45% (14/217).

### Estimation of HIV burden with the two methods

Of the 2.57 million 15–49-year-olds in Guntur district in 2005, 112 635 (4.38%) were estimated to have HIV if the sentinel surveillance method used by NACO was applied, which was 2.5 times the estimate of 45942 (1.79%) based on our population-based study after adjusting for under-represented high-risk groups (Table 9). Sensitivity analysis, using the upper and lower limits of the 95% CIs for the HIV prevalence estimates for men and women in our population-based study, revealed that the ratio of the estimates of people with HIV from the sentinel surveillance and population-based methods could vary from 2.0 to 3.2.

Comparison of the two methods showed that the total number of 15–49-year-olds with HIV in Guntur district based on data from the population-based study including adjustments for people with HIV in the high-risk groups that were under-represented in the population-based sample (45 942) was less than the number calculated for the antenatal data component using the sentinel surveillance method (79 684) even without adding HIV from the STI data component (32008) (Table 9). This STI HIV component of the sentinel surveillance method resulted in an extra 69.7% over the total estimate from the population-based study. Of the 33 742 excess from the antenatal component of the sentinel surveillance method alone compared with the total population-based estimate, 12 749 could be attributed to the over-representation of lower SLI among women using antenatal care in public hospitals in our population-based data (3:1 ratio of lower and upper halves of SLI) and the remaining 20993 to the referral of HIV-positive people to public hospitals, resulting in these two components causing an excess of 27.8% and 45.7% respectively over the total population-based estimate of 45 942.

The total estimate of HIV for the 15–49-year-old population could be arrived at by multiplying the sentinel surveillance HIV prevalence of 3% at the Guntur city antenatal clinic with 0.60 and applying this prevalence to the total population in this age group. Sensitivity analysis, using the upper and lower limits of the 95% CIs for the HIV prevalence estimates for men and women in our population-based study, revealed that this correction factor could range from 0.46 to 0.74. The correction factor would be 0.65 (sensitivity analysis range 0.50–0.81) if the average HIV prevalence of 2.75% from the sentinel surveillance at the Guntur city and Narsaraopet town antenatal clinics was used.

## Discussion

The dramatically lower estimate of the number of people with HIV based on our population-based study than that obtained using the sentinel surveillance data and methods prompted us to examine possible reasons for an underestimation in our population-based study and calculations.

The sample that we selected to represent the 15–49-year-old population of Guntur district was based on a stratified random strategy that does not seem to have any known biases. The major known groups at possibly higher risk of HIV were represented adequately in our sample, except for female sex workers, prisoners and residents of hostels, which we added to our estimate of the number of people with HIV. Male clients of female sex workers were represented in our sample, making up 18.7% of men. Men who reported having sex with men made up 2.1% of our sample of men, and based on other available data from Andhra Pradesh [28,29], this sample had an adequate representation of men who sell sex to men. Homeless people were represented in the sample and their contribution calculated according to their proportion in the population. Young adults living alone or with others of the same sex were represented in our sample. The participation rates of people having transport-related occupations, unskilled labourers and other occupations involving regular mobility, as well as different types of marital status, were similar to the overall participation rates in our study. The urban sample was stratified for the distribution of the different socioeconomic strata, which would be expected to include lower socioeconomic strata including migrant labourers in proportion to their representation in the population, and the proportion of scheduled castes and scheduled tribes (a surrogate measure for lower socioeconomic status) was not under-represented in our rural sample. Finally, we assumed that the men missing in our sample (the proportion of men undersampled compared with their ratio to women in the census data), had twice the prevalence of HIV in sampled men, and added this to our total estimate of HIV to guard against underestimation. We therefore could not identify any reason for a significant underestimation of HIV due to the sampling or calculation method that we used.

We used standard field and laboratory procedures for dried blood spots that have been described in the literature [18-22]. The dried blood spots were stored in the field office in sealed polythene bags with desiccant at room temperature for a maximum of a week, after which they were stored in the laboratory under refrigeration. The time lag between sample collection and laboratory analysis in our study was within the range that has been reported in the literature as not resulting in loss of sensitivity in detecting HIV [21,22]. The laboratory procedures that we used to detect HIV antibody, antigen and nucleic acid from dried blood spots have been successfully used by others previously [20,21]. Our own laboratory comparison of detection of HIV antibody from dried blood spots versus venous blood showed complete concordance. Our quality assurance repeat testing of 10% of the HIV negative samples with fourth-generation ELISA and PCR revealed no false negatives. Although it is possible that a minimal loss of sensitivity in detecting HIV may still have occurred, we could not identify any reason for a significant underestimation of HIV due to technical reasons related to the samples or laboratory analysis.

On the other hand, analysis of our population-based data and its comparison with the sentinel surveillance method used by NACO showed two clear sets of reasons for overestimation of HIV by the latter: (i) inclusion of HIV estimation from STI clinics as a surrogate measure for hidden high-risk groups, and (ii) the profile of women using antenatal care at the public-sector hospitals that are included in the sentinel surveillance method.

Firstly, our analysis showed that in the NACO method, the HIV estimate component from the STI clinic data was not needed, as the HIV estimate component from the antenatal clinic data alone substantially exceeded the population-based estimate for Guntur district even after adjusting for under-represented high-risk groups (Table 9). The STI component was originally included in the NACO method with the assumption that hidden high-risk groups, particularly among men, may not be reflected in the antenatal HIV component. However, our population-based data suggest otherwise. The STI component was a major contributor to the HIV overestimation with the NACO method in our comparison, causing an excess of 70% over our total population-based estimate. The location of sentinel surveillance STI clinics, mostly at large medical college or district headquarter hospitals, makes the magnitude of this overestimation very high, as these clinics get patients with advanced STI, often by referral, who are likely to have a very high risk of HIV [30], as is evident from the median HIV prevalence of 22.8% in these clinics in the sentinel surveillance of 2005 in Andhra Pradesh.

Secondly, our data revealed that only 21% of the women in Guntur district used antenatal care at the public-sector hospitals that are included in the sentinel surveillance, and that the representation of lower SLI was disproportionately higher in this group (Tables 5). We found that in Guntur district, women in the lower SLI half had a HIV prevalence over twice that of the upper half (Table 3). In addition, and more important, even for the same category of SLI, we found much higher HIV prevalence in women in Guntur district using antenatal care at public-sector hospitals compared with those who preferred other options (Table 6). It should be noted though that the confidence intervals for this comparison were wide because the number of HIV-positive women who had been pregnant in the past 2 years was small in our sample, which is a limitation. However, a similar trend was also seen for general health services with much larger numbers in our sample, showing a consistently higher HIV prevalence in both men and women who used the public health system versus those who did not. This observed trend is quite plausible as it can be related to the widely observed practice of private practitioners moving HIV-positive and more complicated patients to public hospitals, as they generally prefer to avoid dealing with such cases. This practice is believed to be quite common in India. There was clear evidence in our data of referral of HIV-positive women to the Guntur antenatal clinic from the private sector. In addition, our data showed that many women were referred to public antenatal clinics for unspecified blood tests, which would probably include the need for HIV testing, if these women were assessed by the private practitioners to be at high risk and therefore undesirable clients, without revealing this to these patients.

It is important to note that the population-based data from Guntur district showed that the HIV prevalence among women who were pregnant during the past 2 years (1.67%) was almost the same as the prevalence in all 15–49-year-old women (1.70%, 95% CI 1.36–2.04%) or the entire population in this age group (1.72%, 95% CI 1.35–2.09%). Addition of under-represented high-risk groups increased the population HIV prevalence only minimally, by 0.07% to 1.79% (Table 9). Therefore, the HIV prevalence among all pregnant women would be a good surrogate for the population prevalence, with only minimal adjustment needed for under-represented high-risk groups. However, owing to certain characteristics of women using antenatal care at the large public-sector hospitals included in the annual sentinel surveillance, the HIV prevalence among them was much higher: 3% in the sentinel surveillance at the Guntur antenatal clinic and 2.95% (95% CI 2.63–3.27%) among 10504 women at this clinic who used PMTCT services in a year (96.9% of all new antenatal registrants). This was due to referral and gravitation of HIV-positive and HIV-suspected women to public hospitals and to a disproportionately higher representation of women with lower SLI, with these two factors causing an excess of 46% and 28%, respectively, in the HIV burden over our total population-based estimate.

Table 10 summarises the methods and findings from previous studies in India that attempted comparison of population-based HIV prevalence with antenatal surveillance prevalence, which include two published studies from Tamil Nadu state and one study from Karnataka state in India for which these comparison data are not published [7,8,31,32]. As this information reveals, serious limitations related to inadequate power due to small sample size, bias in sampling methodology and poor participation rate make it difficult to draw reliable conclusions about this comparison from these studies.

We did not find a significant difference in the HIV prevalence between men and women or between rural and urban residents in Guntur district. With the observed population-based HIV prevalence and cluster design effects, our sample size had 85% power at the 95% confidence level to detect a 45% difference in HIV prevalence between men and women and a 50% difference in HIV prevalence between rural and urban residents [12,13]. The lack of these differences in our study cannot be explained by the minor undersampling of men (3.6% compared with their ratio to women in the census data) or the slightly lower participation rate of urban residents (89.4% compared with 93% for rural residents).

In our study, there was a higher prevalence of HIV among people having a lower SLI, consistently for men and women and for rural and urban residents. To what degree this is due to a higher susceptibility of those with lower SLI to HIV or due to impoverishment of those with HIV needs to be understood further. It would be useful to study this association in other parts of India also. The peaking of the HIV prevalence among women in their late 20s compared with the peak in the 30s in men found in our population-based study is consistent with an earlier age of marriage and sexual debut for women compared with men in India.

The calculations presented by us for the number of people with HIV do not include children or people older than 49 years. Obviously, HIV estimation and its control are also important in these groups. However, because the prevalence of HIV in children and older adults is estimated to be much lower than in 15–49-year-old adults, the sample sizes required for reliable estimation in these groups in population-based studies would be very large. Therefore, this was beyond the scope of our study. Recent UNAIDS estimates suggest that of the total 38.6 million people having HIV globally in 2005, 6% were children ≤ 14 years of age and 7% were ≥ 50 years of age [1].

Use of data from population-based HIV surveys to adjust surveillance data for population estimates has been discussed previously for sub-Saharan Africa [33-35], but such adjustment approaches have not been investigated in India, where the dynamics of HIV distribution and pattern of health service use could be different. As a reliable estimation of HIV burden is a critical first step for informed planning of HIV control and treatment [1,4,5], well-designed and strategically planned population-based studies of the distribution of HIV in the India are essential at 3–4 year intervals to provide reliable estimates and to suggest correction factors that could be applied to the surveillance HIV data in the period between the population-based studies. In addition, it would be useful for such studies to include methodical assessment of risk factors for HIV to better understand the evolving dynamics of HIV in the population.

## Conclusion

This population-based study in a district in south India with relatively high HIV prevalence revealed that the currently used official HIV estimation method in India, which is based on sentinel surveillance data from large public-health hospitals, leads to a 2–3 times higher estimate of HIV burden in this district compared with the population-based estimate adjusted for under-represented high-risk groups. The reasons for overestimation of the HIV burden by the official method, in the order of importance, were: (i) addition of substantial extra HIV estimates from STI clinics; (ii) the common practice of referral of HIV-positive and HIV-suspected people by private practitioners to public hospitals, including antenatal clinics; and (iii) a preferential use of public hospitals by lower socioeconomic strata that had a higher HIV prevalence in this study. The potential major implications of these findings for the overall HIV estimate for India need to be examined.

## Competing interests

The author(s) declare that they have no competing interests.

## Authors' contributions

LD conceived this study, led the overall design and analysis, and drafted the manuscript. VL contributed to the design, led the laboratory analysis, and contributed to the interpretation. TS contributed to the planning of laboratory analysis, performed the HIV testing, and contributed to the interpretation. GAK contributed to the statistical analysis and interpretation. RD contributed to the design, led monitoring of the field study, and contributed to the analysis and interpretation. All authors approved the final version of the manuscript.

## Pre-publication history

The pre-publication history for this paper can be accessed here:



## Figures and Tables

**Table 1:** Estimation of HIV burden by NACO in people 15–49 years of age in Andhra Pradesh, based on sentinel surveillance data for 2005

Population category	Sentinel surveillance based calculations by NACO*		
	
	2005 population (15–49 years)	HIV prevalence†	Number with HIV
Urban men	6 131 043‡	0.0240	147 145
Urban women	5 881 092‡	0.0200	117 622
Rural men	14 464 583‡	0.0240	347 150
Rural women	14 178 474‡	0.0200	283 569
Urban men with STI	391 343§	0.2280	89 226
Urban women with STI	340 978§	0.1900	64 786
Rural men with STI	972 539§	0.2280	221 739
Rural women with STI	870 077§	0.1900	165 315
Female sex workers	117 635¶	0.1280	15 057
Men who have sex with men	5 082**	0.0645	328
Total	43 352 845	0.0335	1 451 937

*These calculations carried out by NACO.

†HIV prevalence from the sentinel surveillance of 2005 in Andhra Pradesh applied as follows [2,6]: median HIV prevalence of 2% from 23 antenatal sentinel surveillance clinics at medical colleges or district headquarter hospitals (sample 400 at each clinic, range of HIV prevalence 0.25–3.5%) applied to urban and rural women 15–49 years old, and 20% higher prevalence than this applied to urban and rural men; median HIV prevalence of 22.8% from 11 STI sentinel surveillance clinics mostly at medical colleges or district headquarter hospitals (sample 250 at 10 clinics and 219 at one clinic, range of HIV prevalence 4–32.4%) applied to urban and rural men assumed to get STI annually, and 83.3% of this prevalence applied to urban and rural women assumed to get STI annually; median HIV prevalence of 12.8% from seven sentinel surveillance sites for female sex workers (sample 250 each, range of HIV prevalence 5.2–26.8%); HIV prevalence of 6.45% from one sentinel surveillance site for men who have sex with men (sample 217); the total HIV prevalence of 3.35% in people 15–49 years of age calculated by dividing the total number calculated to have HIV by the total population in this age group.

‡Excludes men and women shown separately in other categories below.

§In this method, 6% urban men and women and 6.3% rural men and women were assumed to get STI annually; this portion was assumed to cover high-risk groups in the population, excluding female sex workers and men who have sex with men, shown separately [3,6].

¶0.55% of urban and rural women 15–49 years of age, comprising that portion of female sex workers not expected to be covered in the STI component of the calculations [6].

**Number of men who have sex with men estimated in Andhra Pradesh who were not expected to be covered in the STI component of the calculations.

**Table 2:** Population-based sex and rural/urban prevalence of HIV in Guntur district

	Men				Women				Total	
	
	Participants (*n*)	HIV-positive (*n*)	Adjusted HIV prevalence* (%)	95% CI (design effect)	Participants (*n*)	HIV-positive (*n*)	Adjusted HIV prevalence* (%)	95% CI (design effect)	Adjusted HIV prevalence* (%)	95% CI (design effect)
Rural	3139	54	1.57	0.86–2.28 (2.36)	3178	50	1.71	1.18–2.24 (1.43)	1.64	1.10–2.18 (2.86)
Western region (Durgi)	733	6	0.81		763	7	0.97		0.89	0.06–1.72 (3.02)
Central region†	1527	38	2.58		1543	26	1.79		2.19	1.41–2.97 (2.30)
(Mupalla)	757	21	2.73		776	15	2.03		2.39	1.19–3.59 (2.47)
(Narsaraopet)	770	17	2.43		767	11	1.54		1.99	0.92–3.06 (2.36)
Eastern region (Kollur)	879	10	1.23		872	17	1.94		1.58	0.45–2.71 (3.75)
Urban	3096	76	2.11	1.50–2.72 (1.37)	3204	61	1.67	1.22–2.12 (1.04)	1.89	1.39–2.39 (2.13)
Guntur	2358	61	2.45		2443	44	1.63		2.04	1.44–2.64 (2.29)
Narsaraopet	738	15	1.91		761	17	1.83		1.87	0.95–2.79 (1.84)
Total	6235	130	1.74	1.27–2.21 (1.80)	6382	111	1.70	1.36–2.04 (1.21)	1.72	1.35–2.09 (2.44)

*Adjustments explained in the methods section.

†Central region rural population was represented by Mupalla and Narsaraopet mandals as explained in the methods section.

CI, confidence interval.

**Table 3:** Distribution of HIV by standard of living index (SLI) quartiles in the population-based sample

	Men		Women		Men and women	
	
SLI quartiles* (score range)	Participants (*n*)	HIV-positive, *n *(%)	Participants (*n*)	HIV-positive, *n *(%)	Participants (*n*)	HIV-positive, *n *(%)
Rural						
I (up to 16)	1074	18 (1.68)	1140	31 (2.72)	2214	49 (2.21)
II (17–22)	894	22 (2.46)	872	11 (1.26)	1766	33 (1.87)
III (23–29)	745	9 (1.21)	733	5 (0.68)	1478	14 (0.95)
IV (30 or more)	425	5 (1.18)	433	3 (0.69)	858	8 (0.93)
Missing data	1	0			1	0
Total	3139	54 (1.72)	3178	50 (1.57)	6317	104 (1.65)
Urban						
I (up to 16)	490	29 (5.92)	565	20 (3.54)	1055	49 (4.64)
II (17–22)	699	19 (2.72)	731	17 (2.33)	1430	36 (2.52)
III (23–29)	784	10 (1.28)	789	12 (1.52)	1573	22 (1.40)
IV (30 or more)	1123	18 (1.60)	1119	12 (1.07)	2242	30 (1.34)
Total	3096	76 (2.45)	3204	61 (1.90)	6300	137 (2.17)
Rural and urban						
I (up to 16)	1564	47 (3.01)	1705	51 (2.99)	3269	98 (3.00)
II (17–22)	1593	41 (2.57)	1603	28 (1.75)	3196	69 (2.16)
III (23–29)	1529	19 (1.24)	1522	17 (1.12)	3051	36 (1.18)
IV (30 or more)	1548	23 (1.49)	1552	15 (0.97)	3100	38 (1.23)
Missing data	1	0			1	0
Total	6235	130 (2.09)	6382	111 (1.74)	12 617	241 (1.91)

*SLI quartiles correspond to the division of score distribution for the total sample into approximately equal quarters. The sizes of quarters ranged between 3051 and 3269 as the scores were whole numbers and each quartile was defined based on the nearest corresponding score. Lower index score corresponds to lower standard of living.

**Table 4:** Distribution of HIV by age in the population-based sample

	Men		Women		Men and women	
	
Age (years)	Participants (*n*)	HIV-positive, *n *(%)	Participants (*n*)	HIV-positive, *n *(%)	Participants (*n*)	HIV-positive, *n *(%)
Rural						
15–19	595	1 (0.17)	595	8 (1.34)	1190	9 (0.76)
20–24	542	6 (1.11)	562	9 (1.60)	1104	15 (1.36)
25–29	477	10 (2.10)	508	12 (2.36)	985	22 (2.23)
30–34	412	9 (2.18)	451	8 (1.77)	863	17 (1.97)
35–39	449	13 (2.90)	451	8 (1.77)	900	21 (2.33)
40–44	330	8 (2.42)	370	4 (1.08)	700	12 (1.71)
45–49	334	7 (2.10)	241	1 (0.41)	575	8 (1.39)
Total	3139	54 (1.72)	3178	50 (1.57)	6317	104 (1.65)
Urban						
15–19	580	2 (0.34)	564	2 (0.35)	1144	4 (0.35)
20–24	544	10 (1.84)	600	16 (2.67)	1144	26 (2.27)
25–29	495	8 (1.62)	519	14 (2.70)	1014	22 (2.17)
30–34	421	24 (5.70)	435	11 (2.53)	856	35 (4.09)
35–39	393	22 (5.60)	413	9 (2.18)	806	31 (3.85)
40–44	298	7 (2.35)	387	4 (1.03)	685	11 (1.61)
45–49	365	3 (0.82)	286	5 (1.75)	651	8 (1.23)
Total	3096	76 (2.45)	3204	61 (1.90)	6300	137 (2.17)
Rural and urban						
15–19	1175	3 (0.26)	1159	10 (0.86)	2334	13 (0.56)
20–24	1086	16 (1.47)	1162	25 (2.15)	2248	41 (1.82)
25–29	972	18 (1.85)	1027	26 (2.53)	1999	44 (2.20)
30–34	833	33 (3.96)	886	19 (2.14)	1719	52 (3.03)
35–39	842	35 (4.16)	864	17 (1.97)	1706	52 (3.05)
40–44	628	15 (2.39)	757	8 (1.06)	1385	23 (1.66)
45–49	699	10 (1.43)	527	6 (1.14)	1226	16 (1.31)
Total	6235	130 (2.09)	6382	111 (1.74)	12617	241 (1.91)

**Table 5:** Type of antenatal care services used by standard of living index (SLI) quartiles in the population-based sample

		SLI quartiles (score range)			
		
Antenatal care services used	Total	I (up to 16), *n *(%)	II (17–22), *n *(%)	III (23–29), *n *(%)	IV (30 or more), *n *(%)
Public-sector hospitals*	152	68 (44.7)	46 (30.3)	28 (18.4)	10 (6.6)
Other options†	570	144 (25.3)	140 (24.6)	159 (27.9)	127 (22.3)
Private health facility	512	112 (21.9)	121 (23.6)	153 (29.9)	126 (24.6)
Other public-sector facilities	14	6 (42.9)	4 (28.6)	4 (28.6)	0
Checkup at home	8	7 (87.5)	1 (12.5)	0	0
No checkup	36	19 (52.8)	14 (38.9)	2 (5.6)	1 (2.8)
Data not available	55	15 (27.3)	24 (43.6)	11 (20)	5 (9.1)
Total	777	227 (29.2)	210 (27.0)	198 (25.5)	142 (18.3)

*This category includes public-sector hospitals that are covered by sentinel surveillance.

†This category includes options that are not covered by sentinel surveillance, including small public-sector health facilities such as primary health centres and subcentres.

**Table 6:** HIV prevalence by standard of living index (SLI) and type of health services used in the population-based sample

Type of health services used	SLI halves (score range)			
	
	Lower (up to 22)		Upper (23 or more)	
	
	*n*	HIV-positive (%)	*n*	HIV-positive (%)
Antenatal care by women				
Public-sector hospitals*	114	5 (4.39)	38	1 (2.63)
Other†	284	3 (1.06)	286	3 (1.05)
Total	398	8 (2.01)	324	4 (1.23)
General health services by men‡				
Public-sector hospitals*	325	18 (5.54)	165	4 (2.42)
Other†	2831	70 (2.47)	2909	38 (1.31)
Total	3156	88 (2.79)	3074	42 (1.37)
General health services by women‡				
Public-sector hospitals*	516	19 (3.68)	246	4 (1.63)
Other†	2792	60 (2.15)	2827	28 (0.99)
Total	3308	79 (2.39)	3073	32 (1.04)

*This includes public-sector hospitals covered by sentinel surveillance.

†Other comprises mainly of private sector services, and for antenatal care includes a small proportion of smaller public-sector health facilities not covered by sentinel surveillance (total 14 women).

‡Data on five men and one woman missing for type of general health services used, and data on type of public-sector facility used for general health services not available.

**Table 7:** Standard of living index (SLI) distribution of public-sector antenatal clinic attendees

SLI quartiles* (score range)	Public-sector antenatal clinic attendees					
	
	Guntur city			Narsaraopet town		
	
	Rural, *n *(%)	Urban, *n *(%)	Total, *n *(%)	Rural, *n *(%)	Urban, *n *(%)	Total, *n *(%)
I (up to 16)	125 (53.2)	108 (42.9)	233 (47.8)	181 (58.2)	47 (51.6)	228 (56.7)
II (17–22)	69 (29.4)	87 (34.5)	156 (32.0)	63 (20.3)	21 (23.1)	84 (20.9)
III (23–29)	30 (12.8)	49 (19.4)	79 (16.2)	49 (15.8)	19 (20.9)	68 (16.9)
IV (30 or more)	11 (4.7)	8 (3.2)	19 (3.9)	18 (5.8)	4 (4.4)	22 (5.5)
Total	235 (100)	252 (100)	487 (100)	311 (100)	91 (100)	402 (100)

*SLI quartiles based on the distribution of scores in our population-based sample.

**Table 8:** Patient referrals to public-sector antenatal clinics

	Public-sector antenatal clinic attendees					
	
	Guntur city			Narsaraopet town		
	
	Rural, *n *(%)	Urban, *n *(%)	Total, *n *(%)	Rural, *n *(%)	Urban, *n *(%)	Total, *n *(%)
Total	235 (100)	252 (100)	487 (100)	311 (100)	91 (100)	402 (100)
Visited private health facility previously for this pregnancy	95 (40.4)	83 (32.9)	178 (36.6)	112 (36.0)	28 (30.8)	140 (34.8)
Referred by private facility to public-sector hospital for this pregnancy	37 (15.7)	16 (6.3)	53 (10.9)	63 (20.3)	8 (8.8)	71 (17.7)

**Table 9:** Estimation of HIV burden in Guntur district using the two methods

Population category	Sentinel surveillance method			Population-based study method		
	
	2005 population (15–49 years)	HIV prevalence*	Number with HIV	2005 population (15–49 years)	HIV prevalence†	Number with HIV
Urban men	370 005‡	0.0360	13 320	373 926††	0.0210	7 852
Urban women	366 235‡	0.0300	10 987	378 557‡‡	0.0162	6 133
Rural men	848 207‡	0.0360	30 535	873 893§§	0.0157	13 720
Rural women	828 055‡	0.0300	24 842	880 365¶¶	0.0170	14 966
Urban men with STI	23 526§	0.2280	5 364			
Urban women with STI	21 234§	0.1900	4 034			
Rural men with STI	56 821§	0.2280	12 955			
Rural women with STI	50 815§	0.1900	9 655			
Female sex workers	7 003¶	0.1320	924	9 420***	0.1320	1 243
Men who have sex with men	300**	0.0645	19			
Prisoners: men				800†††	0.1050	84
Hostel residents: men				5 000‡‡‡	0.0420	210
Hostel residents: women				5 000‡‡‡	0.0324	162
Undersampled urban men				14 297§§§	0.0420	600
Undersampled rural men				30 943§§§	0.0314	972
Total	2 572 201	0.0438	112 635	2 572 201	0.0179	45 942¶¶¶

*HIV prevalence used in the sentinel surveillance method: HIV prevalence from the antenatal sentinel surveillance of 2005 in Guntur at the medical college clinic applied to urban and rural women 15–49 years old, and 20% higher prevalence than this applied to urban and rural men; 2005 HIV prevalence from the sentinel surveillance STI clinic applied to urban and rural men assumed to get STI annually, and 83.3% of this prevalence applied to urban and rural women assumed to get STI annually; 2005 HIV prevalence from the sentinel sites for female sex workers and men who have sex with men used [6].

†HIV prevalence used in the population-based study method: HIV prevalence for urban and rural men and women used from our population-based study; HIV prevalence in men in prisons assumed as 5 times that in urban men; HIV prevalence in men and women hostel residents assumed as twice the urban prevalence in each sex; HIV prevalence in undersampled urban and rural men assumed as twice the prevalence in sampled urban and rural men.

‡Excludes men and women shown separately in other categories below.

§In this method, 6% urban men and women, and 6.3% rural men and women were assumed to get STI annually; this portion assumed to cover high-risk groups in the population, excluding female sex workers and men who have sex with men, shown separately [3,6].

¶0.55% of urban and rural women comprising that portion of female sex workers not expected to be covered in the STI component of the calculations [6].

**This method estimated 5082 men who have sex with men in Andhra Pradesh who were not expected to be covered in the STI component of the calculations (Table 1); for Guntur district we used the fraction proportional to its 5.9% population contribution to the state.

††Excludes urban men in prisons at a given time, residents of hostels, and undersampled urban men.

‡‡Excludes urban female sex workers estimated not covered in our population-based sample and residents of hostels.

§§Excludes rural men in prisons at a given time and undersampled rural men.

¶¶Excludes rural female sex workers estimated not covered in our population-based sample.

***Our urban and rural women samples included five (0.14%) and four (0.12%) women, respectively, who were identified as sex workers, although other unidentified sex workers would also likely be part of our sample; Guntur district was estimated to have 11000 female sex workers in 2005 [9], 0.86% of women in the 15–49-year age group; based on our previous study of female sex workers in Andhra Pradesh [26,27], we extrapolated that 60% sex workers are in urban areas and 40% in rural areas, suggesting that in Guntur district 6600 sex workers would be urban and 4400 rural; this implied that 6055 urban and 3365 rural sex workers were not represented in our population-based sample, and are therefore shown separately.

††Estimated based on capacity of prisons in Guntur district, and assumed that half the prisoners are urban and half are rural.

‡‡We estimated by performing a census of residential hostels for students and working people in the urban areas of Guntur district that 5000 men and 5000 women 18–29 years old, and 7000 boys and 3500 girls 15–17 years old, would be residing in these hostels. The younger age group comprises of students mostly in strictly supervised residential schools, and therefore, their risk of HIV was considered similar to that in the general population; the 18–29-year age group comprises of college students and working people living mostly without any supervision, and therefore, the HIV prevalence for them was considered twice the urban prevalence for each sex. As the estimated number in this latter category was the same for men and women, and in India more young men than women would be expected to be living alone or with others of the same sex, we assessed our sample for the number of 18–29-year-old men and women living alone or with others of the same sex outside the hostel setting and found 76 men (5.8% of urban men sample in this age group) and 14 (1% of urban women sample in this age group) in this category.

§§§Our population-based sample had 3.7% and 3.5% undersampling of urban and rural men, respectively, compared with their ratio to women in the Census data [23]; we considered this undersampled group separately, after excluding prisoners, assuming that these missing men would be at higher risk of HIV.

¶¶¶Some other groups considered at relatively high risk of HIV, men who have sex with men (including men who sell sex to men), intravenous drug users, and migrant labourers, were not added separately to this calculation for the following reasons. (i) Our population-based sample had 2.1% men who reported having had sex with men, which included 0.26% men who had sold sex to men. In our previous study of 6661 men who had sex with men recruited through extensive snowball sampling in 13 districts of Andhra Pradesh 26.7% had sold sex to men [28,29], which was 0.015% of 15–49-year-old men in these 13 districts; this fraction was 17 times less than the fraction of 0.26% in our population-based sample. The latter would seem to be an adequate representation of this group in our sample if we conservatively assume that we had actually reached only 1/17th of the actual men who sell sex to men in our extensive snowball sampling method in the 13-district study. (ii) The number of intravenous drug users is considered to be negligible in Andhra Pradesh. (iii) We estimated that our stratified random sampling strategy, which had adequate coverage of lower socioeconomic strata, would include migrant labourers residing in the sampled areas for 6 months or more according to their proportion in the population; even if this were not completely so, the addition of undersampled men with assumed higher HIV prevalence to the calculation, as mentioned above, would cover this group.

**Table 10:** Methods and findings from studies comparing population-based and sentinel surveillance antenatal HIV prevalence in India.

Location [Reference]	Data collection year(s)	Sampling approach	Participation rate	Sample size (*n*)	Number of HIV-positive	Population HIV prevalence as % (95% CI)*	Power of sample size to detect difference from antenatal HIV prevalence at 95% confidence level†	Comments
Tamil Nadu: 3 districts [7]	1998	90 rural & urban clusters selected using probability proportional to size; selected households from each cluster invited for medical camp; first 25 adults 15–45 years old from each cluster who came to camp included in study	82.5% for selected households; not mentioned for eligible individuals	1981	34	Age & sex adjusted: 1.80 (0.89–2.71)	17% to detect 20% difference from 1% antenatal HIV prevalence	Selection bias likely due to medical camp sampling approach, making interpretation difficult; Grossly underpowered for reliable comparison with antenatal HIV prevalence
Tamil Nadu: 1 rural sub-district, 1 urban town [8]	1999–2000	120 rural & urban clusters selected using probability proportional to size; 15–40 years old people from randomly selected households included in study	90.9% of 3–40-year-olds; not mentioned for eligible 15–40-year-olds	2870	29	Crude: 1.01 (0.44–1.58)	21% to detect 20% difference from 1% antenatal HIV prevalence	Grossly underpowered for reliable comparison with antenatal HIV prevalence
Karnataka: 1 district [31,32]	2003	10 villages and 20 urban blocks selected with cluster sampling using probability proportional to size; 15–49-year-olds included in study; further details not published	59.8% of 6700 eligible 15–49-year-olds	4008	118	Crude: 2.94 (2.12–3.76)	50% to detect 20% difference from 2.6% antenatal HIV prevalence	Poor participation rate makes interpretation difficult; Underpowered for reliable comparison with antenatal HIV prevalence
Andhra Pradesh: 1 district [This study]	2004–2005	5 subdistricts selected to represent strata in district, from which 66 rural & urban clusters selected randomly; 15–49-year-olds from randomly selected households included in study	91.2% of 13838 eligible 15–49-year-olds	12617	241	Age, sex & rural-urban adjusted: 1.72 (1.35–2.09)	93% to detect 20% difference from 3% antenatal HIV prevalence	Adequately powered for reliable comparison with antenatal HIV prevalence

*Although the two Tamil Nadu papers reported adjusting for cluster design effect, the magnitude of this effect was not reported, and the confidence intervals reported in both these papers are implausibly narrow even if no design effect were considered (cluster design effect widens the confidence interval). The Karnataka study did not report design effect information. Because specific details about cluster design effect in these studies were not available, we used the cluster design effect of 2.44 from our study to calculate the confidence intervals for the other studies, using standard statistical methods [12,24]

†Power calculated assuming cluster design effect of 2.44 for all studies, using standard statistical methods [12,13]; sentinel surveillance antenatal HIV prevalence for comparison as reported in each study.
